# Surgical versus Nonsurgical Multimodality Treatment in an Idiopathic Frozen Shoulder: A Retrospective Study of Clinical and Functional Outcomes

**DOI:** 10.3390/jcm10215185

**Published:** 2021-11-05

**Authors:** Wojciech Satora, Roman Brzóska, Robert Prill, Paweł Reichert, Łukasz Oleksy, Anna Mika, Aleksandra Królikowska

**Affiliations:** 1 Department of Orthopaedics, St Luke’s Hospital, 43-309 Bielsko-Biała, Poland; satorawojciech@gmail.com (W.S.); rbrzoska13@gmail.com (R.B.); 2 Center of Orthopaedics and Traumatology, University of Brandenburg an der Havel Theodor Fontane, 14770 Brandenburg an der Havel, Germany; Robert.Prill@mhb-fontane.de; 3 Department of Trauma and Hand Surgery, Faculty of Medicine, Wroclaw Medical University, 50-367 Wroclaw, Poland; pawel.reichert@umw.edu.pl; 4 Orthopaedic and Rehabilitation Department, Medical University of Warsaw, 02-091 Warsaw, Poland; loleksy@oleksy-fizjoterapia.pl; 5 Physiotherapy and Sports Centre, Rzeszow University of Technology, 35-959 Rzeszów, Poland; 6 Institute of Clinical Rehabilitation, University of Physical Education in Kraków, 31-571 Kraków, Poland; anna.mika@awf.krakow.pl; 7 Ergonomics and Biomedical Monitoring Laboratory, Department of Physiotherapy, Faculty of Health Sciences, Wroclaw Medical University, 50-367 Wroclaw, Poland

**Keywords:** adhesive capsulitis, arthroscopy, corticosteroids, frozen shoulder, physiotherapy, physical therapy, Quick DASH, rehabilitation, surgical treatment

## Abstract

This retrospective study compared the clinical and functional outcomes of patients diagnosed with an idiopathic frozen shoulder with symptom onset of a maximum of six months, treated by arthroscopic capsular release followed by corticosteroid injection and physiotherapy to patients who received only corticosteroid injection followed by physiotherapy. The patients who underwent arthroscopic capsular release, intraoperative corticosteroid injection, and physiotherapy (Group I, *n* = 30) or received only corticosteroids injection and physiotherapy (Group II, *n* = 29) were examined in terms of shoulder range of motion (ROM), pain intensity, and function before a given treatment and three, six, and twelve months later. The groups were comparable pre-treatment in terms of ROM, pain, and functional outcome. Group I had statistically and clinically significantly better ROM and function at three and six months post-treatment than Group II. Despite being statistically significant, the between-group differences at twelve-month follow-up in ROM and function were too small to be considered clinically notable. The between-group comparison of pain revealed no significant differences at any post-treatment point of time. The early arthroscopic capsular release preceding corticosteroid injection and physiotherapy seemed more effective at three- and six-month follow-up; however, it brought a comparable result to corticosteroid injection and subsequent physiotherapy at twelve months follow-up.

## 1. Introduction

Although a prevalence of 2% to 5% in the general population makes frozen shoulder one of the most common causes of shoulder pain and disability in the upper extremity [1], the best option for its treatment remains debatable. This pathological process appears to start as an inflammatory reaction in the capsule with associated synovitis that progresses to the fibrotic contracture of the capsule [2,3,4]. The onset of the disease is reported as shoulder pain, followed by restriction of shoulder mobility, most commonly forward flexion, abduction, and external rotation [5,6].

The therapy aims to regain a painless and functional shoulder with the full range of motion. Routinely, the first choice of intervention is a nonsurgical one, represented by pharmacotherapy of the synovitis and inflammatory mediators and/or physiotherapy treatment modalities to prevent or modify capsular contracture [7,8,9]. Hydrodilatation [10] and platelet-rich plasma injections [11] have also been indicated as common therapeutic options [11]. The surgical treatment, which consists of manipulation under anesthesia and arthroscopic capsular release, treats both the inflammatory component via synovectomy and the capsular contracture through manipulation under anesthesia [12,13].

However, the optimal time to perform operative treatment in patients with frozen shoulder remains controversial. Surgical intervention in the early stages is still a relatively rarely handled topic [14,15,16]. Primarily, this has been because the disease has always been considered mild, with symptoms resolving spontaneously sooner or later [17]. Besides, it has been suggested that early surgery leads to a poor prognosis. Another critical issue is possible postoperative complications, which should always be considered when going for surgical intervention [18].

On the other hand, it has been indicated that some patients are seeking a quicker resolution of symptoms and are willing to undergo surgery [16], as it should be emphasized that the entire course of the disease may last for even two or three years [19]. Symptoms may persist long term, significantly affecting daily living activities and impairing quality of life [20].

Recent studies showed that arthroscopic capsular release performed less than six months after the onset of frozen shoulder symptoms does not provide a poor prognosis than delayed arthroscopy [14]. Nonetheless, it has been highlighted that there is a lack of studies comparing early surgical intervention outcomes with nonsurgical procedures [21].

The study aimed to compare short- and middle-term clinical and functional outcomes of patients diagnosed with an idiopathic frozen shoulder with symptom onset of a maximum of six months treated by arthroscopic capsular release, corticosteroid injection, and subsequent postoperative physiotherapy to patients who received corticosteroid injection followed by the physiotherapeutic procedure.

## 2. Materials and Methods

The study used a retrospective comparative design. The experiment was conducted according to the Declaration of Helsinki’s ethics guidelines and principles and was approved by the Bioethics Committee of the Wroclaw Medical University, Wroclaw, Poland (KB 772/2017). The data gathered in this study are available as a Supplementary Materials file named S1 Database.

### 2.1. Characteristics of the Studied Participants

The initial sample comprised 148 patients diagnosed with a frozen shoulder and consecutively treated in 2010–2016 in the Department of Orthopaedics, St Luke’s Hospital, Bielsko-Biała, Poland. The frozen shoulder diagnosis was made based on the patients’ history and physical examination. Due to the retrospective character of the study, to be sure of the decision whether the frozen shoulder was primary or secondary, the initial sample comprised only patients with magnetic resonance imaging to rule out any other causes of a painful, stiff shoulder. The exclusion criteria were bilateral incidence of frozen shoulder (*n* = 33), secondary frozen shoulder (*n* = 50), diagnosed diabetes (*n* = 4), diagnosed thyroid disorder (*n* = 2), diagnosed adrenal disorder, diagnosed cardiovascular disease, and diagnosed hyperlipidemia. The remaining patients were divided into two groups: Group I (*n* = 30) received an early arthroscopic capsular release, corticosteroid injection, and subsequent physiotherapy, and Group II (*n* = 29) received only the corticosteroid injection and physiotherapeutic procedure.

Before group assignment, the patients reported a minimum of three months of unstructured treatment, involving mainly self-administrated nonsteroidal anti-inflammatory drugs. Regarding the choice of treatment, patients were offered to consider surgery as an alternative treatment method. However, it was highlighted that, according to current medical knowledge, there is no conclusive scientific evidence indicating the advantage of surgical treatment over nonsurgical treatment. Patients were also informed about possible complications related to surgical treatment. The final decision about the treatment method was made by the patient.

The studied groups were similar in age (Group I, x = 49.80 ± 2.55 years; Group II, x = 49.69 ± 4.74 years). Women constituted 57% of Group I (*n* = 17) and 69% of Group II (*n* = 20). The time from onset of symptoms that the patients referred to at baseline was comparable in the studied groups (Group I, x = 4.27 ± 1.20 months; Group II, x = 4.07 ± 1.19 months). In all 59 patients, the right upper limb was the dominant one. In Group I, 43% of the involved limbs were right limbs (*n* = 13). In Group II, 48% of the involved limbs were right limbs (*n* = 14).

### 2.2. Surgical Treatment in Group I

Arthroscopic capsular release in patients from Group I was performed by two experienced orthopedic surgeons (R.B. and W.S.). Surgery was conducted in the beach chair position without arm traction. Arthroscopy was started from the posterior portal in a typical way. Arthroscopic optics (4 mm, 30 degree angle, Arthrex, Naples, FL, USA) were inserted through the posterior portal to visualize the anterior part of the joint. Then, in the triangle between the long head of the biceps tendon (LHB), subscapularis tendon (SSC), and the glenoid, the anterior portal was performed under visual control. Subsequently, the anterolateral portal was created above the LHB. After confirming synovial hyperplasia and extensive congestion in all cases, a subsequent synovectomy was performed using a 4.5 mm shaver. It was crucial to identify proper landmarks of rotator interval (RI)—the edge of the supraspinatus tendon to the upper edge of the SSC tendon, coracohumeral ligament (CHL). The CHL was released and separated from the base of the coracoid process, and the whole RI was removed in the first step. Subsequently, the middle glenohumeral ligament was dissected, and the SSC tenolysis was performed, with its separation from the glenohumeral ligaments by electrocautery. The articular capsule was successively released from the base of the coracoid process along the glenoid margin in the posterior direction. Then, the camera was placed on the anterior portal. Under direct visualization, the posterior and inferior capsule were cut by electrocautery inserted through the posterior portal. The remaining adhesions within the articular capsule were released if they were present. After the capsulotomy, mobility of the joint was assessed to ensure that the full range of motion was restored.

After removing the residual saline solution from the joint at the end of the procedure, *Methylprednisoloni acetas* + *Lidocaini hydrochloridum* (40 mg + 10 mg)/mL, namely, 1 mL of Depo-Medrol (Pfizer Inc., New York, NY, USA), was injected.

### 2.3. Postoperative Physiotherapeutic Procedure in Group I

The supervised postoperative physiotherapeutic procedure was focused on increasing the range of passive and active joint ROM, preventing muscular atrophy, and restoring proprioception. The process was based on exercise therapy, including assisted exercises of the involved shoulder, isometric exercises, contralateral and ipsilateral exercises, pendulum exercises, and proprioception exercises. The elements of manual therapy and the proprioceptive neuromuscular facilitation (PNF) method were also used. Furthermore, stretching, including mainly self-stretching, was required. The exercises were performed in the pain-free ROM and introduced sequentially in the sagittal, frontal, transverse, and rotation planes. Consecutively, muscle strength was increased by performing exercises in a closed kinematic chain and then in an open kinematic chain. Patients were also encouraged to do short 10–15 min home exercise sessions several times a day. The whole supervised postoperative procedure lasted for about 16 weeks, with a mean frequency of three visits per week.

### 2.4. Physiotherapeutic Procedure in Group II

Before starting the physiotherapeutic procedure, *Methylprednisoloni acetas* + *Lidocaini hydrochloridum* (40 mg + 10 mg)/mL, namely 1 mL of Depo-Medrol (Pfizer Inc., New York, NY, USA) was injected once into the joint. The ultrasound-guided injections in Group II were performed by the same experienced orthopedic surgeon (R.B.).

The supervised physiotherapeutic procedure was primarily focused on anti-inflammatory and analgesic effects, increasing the range of passive and successively active joint ROM, preventing muscular atrophy, and restoring proprioception. Physical modalities were used, including laser therapy and transcutaneous electrical nerve stimulation. The exercise therapy included passive and assisted exercises of the involved shoulder, isometric exercises, contralateral and ipsilateral exercises, pendulum exercises, and proprioception exercises. Elements of manual therapy, such as traction and elements of the PNF method, starting with patterns for the scapula, were carried out. Auto-stretching was also provided. The exercises were performed painlessly and were introduced sequentially in the sagittal, frontal, transverse, and rotation planes. After restoring the ROM without pain, exercises were performed to increase muscle strength, initially in a closed kinematic chain and consecutively in an open kinematic chain. First, bands with progressive resistance were used, followed by weights and dumbbells. Just as in Group I, the patients were encouraged to do short 10–15 min home exercise sessions several times per day. The whole supervised procedure lasted for about 16 weeks, with a mean frequency of three visits per week.

### 2.5. Outcome Assessment

Outcomes were taken at baseline (just before the given treatment; T0) and consecutively in short-term (three months from baseline; T1), early mid-term (six months from baseline; T2), and late mid-term (twelve months from baseline; T3) follow-ups.

The test procedures included clinical examination based on the ROM measurements, pain assessment, and functional evaluation. Based on the methodology of other studies assessing patients with frozen shoulder [7], the bilateral measurements of passive ROM, including shoulder forward flexion, abduction, external and internal rotation, were carried out using a standard goniometer. The same examiner, an experienced orthopedic surgeon (W.S.), performed all of the ROM measurements. The patient-reported functional evaluation was performed using the Polish version of Quick Disabilities of the Arm, Shoulder and Hand (Quick DASH) [22], with a final score ranging between 0, indicating no disability, and 100 meaning the greatest possible disability. The mean intensity of the daily pain at rest reported by the patient was assessed using the 100 mm Visual Analogue Scale (VAS), a reliable method of evaluating acute and chronic musculoskeletal pain [23,24], with higher scores indicating higher pain intensity. The occurrence of any post-treatment adverse events was documented.

### 2.6. Statistical Analysis

TIBCO Statistica™ (TIBCO Software Inc., Palo Alto, CA, USA) and Microsoft Office Excel 365 Personal (Microsoft Corporation, Redmond, WA, USA) were used for statistical analysis. The number of patients was indicated as *n*. The data collected during the clinical examination and functional evaluation were numerical data. The arithmetic mean (x) and standard deviation (±) were calculated for particular studied features. The Shapiro-Wilk test for normality was used to study the distribution of the features.

The within-group analysis was first based on comparing the ROM values obtained in the involved shoulder to values obtained in the uninvolved shoulder in the particular groups at particular follow-up points. The parametric *t*-test for dependent samples was used for this analysis. Second, in the within-group analysis, the values obtained in the involved limb in a particular group were compared between the consecutive follow-up points using a parametric *t*-test for dependent samples. The same comparison was performed for the uninvolved shoulder. In the between-group statistical analysis, the ROM results in the involved shoulder in one group were compared to the involved limb in the second group at particular time intervals using a parametric *t*-test for independent samples. The ROM values obtained in the uninvolved limbs between the studied groups were compared in the same way. The values of pain intensity on the VAS (mm) and the number of points obtained on the Quick DASH were analyzed within studied groups by comparing the results obtained at consecutive time intervals using a parametric *t*-test for dependent samples. To compare the results between the studied groups at individual time intervals, a parametric *t*-test for independent samples was used. Statistical significance was set at *p* < 0.05. No statistically significant differences were indicated as ns.

Apart from statistical significance, the results were also interpreted in terms of their clinical relevance. Following Challaoumas et al. (2020), the minimal clinically relevant difference amounted to 10° for ROM and 10 mm for VAS [7]. Following Jensen et al. (2003), the VAS ratings of 0.00 to 4.00 mm were considered no pain; 5.00 to 44.00 mm, mild pain; 45.00 to 74.00 mm, moderate pain; and 75.00 to 100.00 mm, severe pain [25]. A 33% decrease in pain represented a reasonable standard for determining that a change in pain is meaningful from the patient’s perspective [25]. The clinical relevance of the obtained Quick DASH scores was also based on the minimal clinically important difference, also known as the minimal important change, representing the smallest improvement in the score that reflects a clinically meaningful change for the patient. Following Franchignoni et al. (2014), the minimal clinically important difference for the Quick DASH amounted to 15.91 points [26].

## 3. Results

### 3.1. Within-Group Comparison

The between-limb comparison of shoulder forward flexion, abduction, and external rotation in Group I revealed statistically significant and clinically relevant worse values in the involved limb compared to the uninvolved limb at T0 and T1, as presented in Table 1. At T2 and T3, the forward flexion, abduction, and external rotation values were statistically significantly lower in the involved shoulder than in the uninvolved one. However, the raw mean differences were too small to be considered clinically meaningful. This was in line with a statistically and clinically significant increase of forward flexion, abduction and external rotation of the involved shoulder at T1 compared to the T0, and at T2 compared to T1. That was also relevant to the lack of statistically or clinically substantial increases of involved shoulder forward flexion, abduction, and external rotation between the T2 and T3. When it comes to involved shoulder internal rotation, the obtained values were statistically and clinically worse comparing to uninvolved shoulder only at T0. The values obtained at T1, T2, and T3 were too small to be considered clinically meaningful, even though they were statistically significant. The clinically meaningful and statistically significant increase of involved shoulder internal rotation was noted at T1 compared to the T0 (*p* ≤ 0.001). At this point, the involved shoulder internal rotation was comparable in terms of clinical meaning to uninvolved shoulder, so no clinically or statistically significant improvements in involved shoulder internal rotation were observed at T2 and T3.

In Group II, the forward flexion, abduction, and external rotation values in the involved shoulder were statistically and clinically worse than in the uninvolved shoulder at T0 and consecutively at T1 and T2 (Table 1). The between-limb differences were also observed at T3. Still, at T3, they were too small to be clinically meaningful. The forward flexion, abduction and external rotation in the involved shoulder gradually, statistically, and clinically significantly increased from the T0 to T1, T2, and T3.

Values expressed as arithmetic mean and standard deviation, ±. As also presented in Table 1, the involved shoulder internal rotation was statistically and clinically worse than in the uninvolved shoulder at T0 and T1. Even though there were noted statistically significant between-limbs differences at T2 and T3, they were not clinically meaningful. The involved shoulder internal rotation had been statistically and clinically increased at T1 compared to T0 and at T2 compared to T1. A statistically significant increase between the T2 and T3 was noted; however, it was not clinically meaningful.

The VAS values obtained were significantly different between the follow-up points in Group I (*p* ≤ 0.001) and Group II (*p* ≤ 0.001), which is presented in Table 2. The pain intensity significantly decreased from moderate to mild between the T0 and T1 in Group I and Group II. It also reduced significantly in both groups and remained mild in subsequent time intervals. The decrease in the mean intensity of pain at T1 compared to T0, as well as at T2 compared to T1, and at T3 comparing with T2 in both studied groups also represented a meaningful change from the patient’s perspective, as it was greater than 33%.

The obtained values of Quick DASH scores significantly differed between the follow-up points in Groups I and II as presented in Table 3. In Group I, the Quick DASH final score was significantly lower (*p* ≤ 0.001) at T1. The improvement in the functional score was also clinically relevant. Although not clinically meaningful, the improvement in functional assessment results between T2 and T1 was statistically significant (*p* ≤ 0.001). The result at T3 was statistically and clinically at the same level as at T2. Furthermore, in Group II, the number of points obtained in Quick DASH was significantly lower at T1 than at T0 (*p* ≤ 0.001). However, the difference was too small to be considered clinically notable. The more considerable improvement in the functional assessment was statistically (*p* ≤ 0.001) and clinically meaningful in Group II between the T1 and T2 months and consecutively between T2 and T3.

In two cases (7%) in Group II, patients underwent arthroscopic capsular release six months from the baseline after an unsuccessful attempt of physiotherapeutic treatment. They did not attend the last examination that took place 12 months after the baseline. Their data were therefore not included in the statistical analysis in the last examination in Group II. No adverse events were noted in Group II.

### 3.2. Between-Group Comparison

At T0, no statistically significant differences were noted between the studied groups regarding involved shoulder flexion, abduction, and external and internal rotation. At T1 and T2, statistically significant and clinically meaningful better values of flexion (*p* ≤ 0.001), abduction (*p* ≤ 0.001), and external rotation (*p* ≤ 0.001) were stated in the involved shoulder in Group I in comparison to the involved shoulder in Group II. However, note that the raw mean differences between the two studied groups were much higher at T1 than at T2. When it comes to involved shoulder internal rotation, the obtained values were statistically (*p* ≤ 0.001) and clinically better at T1 in Group I than in Group II. At T2 and T3, the values were comparable in both groups, and the noted differences were not statistically nor clinically meaningful. At T3, the involved shoulder’s abduction range in Group I was statistically (*p* = ns) and clinically comparable to the range of motion in the involved shoulder in Group II. Even though forward flexion (*p* ≤ 0.001) and external rotation (*p* ≤ 0.001) were statistically significantly better in Group I, the difference was too small to be considered clinically notable. The two studied groups were also comparable in terms of the range of flexion (*p* = ns) and abduction (*p* = ns) of the uninvolved shoulder. There were noted statistically lower external rotation values in the uninvolved shoulder in Group I than in Group II at T0 (*p* = 0.011), T1 (*p* = 0.013), T2 (*p* = 0.015), T3 (*p* = 0.002); however, the difference was too small to be clinically meaningful. The same was for uninvolved shoulder internal rotation, which was statistically significantly larger in Group II than Group I, but the differences were too small to be considered clinically significant.

The between-group comparison of involved limb pain intensity revealed no significant differences at T0 or consecutively at T1, T2, and T3, as presented in Table 2.

Group I and Group II were comparable in functional assessment results at baseline (*p* = 0.615), as presented in Table 3. The most statistically (*p* ≤ 0.001) and clinically notable difference in functional outcome between the studied groups was noted at T1 and T2, favoring Group I. Even though the difference between the two studied groups was statistically significant (*p* = 0.004) at T3, it should not be considered clinically relevant.

## 4. Discussion

Patients with an idiopathic frozen shoulder with symptom onset of a maximum of six months receiving an arthroscopic capsular release, corticosteroid injection, and subsequent physiotherapy showed faster improvement in the involved shoulder’s range of motion and functional outcome than patients who received the corticosteroid injection and consecutive physiotherapeutic procedure. Furthermore, at the early mid-term follow-up point, the arthroscopy had a pronounced effect on range of motion and function. However, the arthroscopic capsular release had no beneficial effect on late mid-term clinical and functional outcomes, as both studied multimodality treatments were successful in that matter. Furthermore, both studied multimodality therapies were equally efficacious at all follow-up points in reducing pain in patients with an idiopathic frozen shoulder.

Despite being relatively common, one might say a frozen shoulder remains full of controversy. One of the debatable issues concerns the best treatment method. Through various nonsurgical procedures remaining a gold standard of treatment, many studies investigated nonsteroidal anti-inflammatory drugs, corticosteroids including oral steroids and local injectable steroids, and physiotherapy [27,28,29,30,31,32,33,34]. There have also been some studies concerning the usage of acupuncture [35,36], hydrodilatation [31,37], calcitonin [38], extracorporeal shock wave therapy [39], and nerve block [34,40]. The surgical treatment that consists of manipulation under anesthesia and arthroscopic capsular release is recommended only when an extended nonsurgical therapy for 6–9 months is unsuccessful [41,42,43].

The justification for waiting to decide on surgical treatment in patients with an idiopathic frozen shoulder is that it has always been considered a disease starting with a decreasing function in the first month in every case, with symptoms resolving spontaneously sooner or later [17]. Some studies report even up to 90% of patients in whom nonsurgical methods or even no therapy is used will resolve the symptoms of the disease [42,44]. The disease’s natural course and pathogenesis are still unknown. However, three consecutive periods can be distinguished from the frozen shoulder: the freezing phase, frozen phase, and thawing phase [45,46]. The disease may last for years, significantly affecting daily living activities and impairing quality of life [20], so it is not surprising that some patients seek a quicker resolution of symptoms and are willing to undergo surgery [16]. For sure, one of the main limitations of the present study is the lack of comparison of patients with an idiopathic frozen shoulder treated with early arthroscopic capsular release and subsequent physiotherapy and patients treated with physiotherapy alone to patients receiving only arthroscopic capsular release and to those not receiving any form of treatment [21].

A second reason for delaying surgical intervention is a common suggestion that early surgery in patients with a frozen shoulder leads to a poor prognosis [14]. Nonetheless, a recently published study showed that surgical intervention performed less than six months after the onset of symptoms does not provide a poorer prognosis than delayed surgical treatment, even though it is related to more severe glenohumeral synovitis than in later surgical intervention [14].

Another critical reason for delaying surgical intervention is the possibility of complications occurring in case of manipulation under anesthesia in at least 0.4% and 14%. The postoperative complications that primarily affect manipulation under anesthesia rather than arthroscopic capsular release include humerus shaft fracture, rotator cuff tear, shoulder dislocation, labral tear, nerve injury, and complex regional pain syndrome [47,48,49,50,51]. In the present study, there were no postoperative complications in the group of patients who underwent early arthroscopic capsular release. It needs to be mentioned that the group of patients was too small to finally conclude that arthroscopy is not related to the risk of postoperative complications. For two patients (7%) in the studied group, the physiotherapeutic procedure alone has been ineffective. Those patients were referred for surgical intervention after the examination taken six months from the baseline. This would be in line with the general assumption that conservative treatment of frozen shoulder is unsuccessful in ~10% of patients [42,44,52], with respect to the idea of six months may still belong to the so-called freezing phase with a natural decrease of ROM and function and increase of pain.

An undoubtedly most significant limitation of the present study is its retrospective design which made the rigor and strength of the randomized trial impossible to provide. The question of early arthroscopic treatment in patients with a frozen shoulder will certainly not be resolved until large prospective randomized clinical trials compare this method of treatment with nonsurgical procedures and with patients who have not received any treatment. In our opinion, besides the need for a better pathophysiological understanding of the disease and its causal cause and development, it also seems necessary to look for improvement and standardization of nonsurgical ways to bring successful and more rapid reduction of symptoms, being not invasive and do not bring the burden of the possibility of postoperative complications.

## 5. Conclusions

Patients diagnosed with an idiopathic frozen shoulder with symptom onset of a maximum of six months receiving arthroscopic capsular release and corticosteroid injection followed by postoperative physiotherapy showed faster improvement in the involved shoulder range of motion and in the functional outcome than patients who received only the corticosteroid injection and physiotherapeutic procedure. Furthermore, at the early mid-term follow-up point, the early arthroscopy had a pronounced effect on range of motion and function. Nonetheless, the arthroscopic capsular release had no beneficial effect on late mid-term clinical and functional outcomes, as both studied multimodality treatments were successful in that matter. Moreover, studied multimodality therapies were equally efficacious in reducing pain in patients with idiopathic frozen shoulders. Therefore, it seems that no recommendation for the early arthroscopic release can be given; however, conclusions should be interpreted with caution, given that they are based on a retrospective analysis.

## Figures and Tables

**Table 1:** The within-group comparison of the obtained values of shoulder range of motion expressed in degrees.

	Follow-Up Point	Group I			Group II		
		Involved Limb	Uninvolved Limb	*p*	Involved Limb	Uninvolved Limb	*p*
Passive shoulder forward flexion	T0	86.67 ± 2.68	163.33 ± 2.60	≤0.001	85.76 ± 3.11	164.48 ± 3.04	≤0.001
	T1	141.33 ± 4.05	163.33 ± 2.60	≤0.001	102.28 ± 3.94	163.69 ± 2.36	≤0.001
	*p*	≤0.001	ns		≤0.001	ns	
	T2	160.63 ± 3.05	163.33 ± 2.60	≤0.001	146.41 ± 15.44	163.69 ± 2.36	≤0.001
	*p*	≤0.001	ns		≤0.001	ns	
	T3	162.53 ± 2.15	163.83 ± 2.57	0.005	160.22 ± 2.99	164.11 ± 3.02	≤0.001
	*p*	ns	ns		≤0.001	ns	
Passive shoulder abduction	T0	68.50 ± 4.45	167.10 ± 3.35	≤0.001	66.90 ± 4.40	167.07 ± 3.95	≤0.001
	T1	129.07 ± 6.54	167.10 ± 3.35	≤0.001	97.90 ± 9.08	167.07 ± 3.95	≤0.001
	*p*	≤0.001	ns		≤0.001	ns	
	T2	164.90 ± 5.01	167.10 ± 3.35	≤0.001	151.93 ± 22.30	167.07 ± 3.95	≤0.001
	*p*	≤0.001	ns		≤0.001	ns	
	T3	164.93 ± 4.66	167.10 ± 3.35	≤0.001	164.04 ± 4.16	167.44 ± 3.49	≤0.001
	*p*	ns	ns		0.010	ns	
Passive shoulder external rotation	T0	11.60 ± 2.71	63.33 ± 2.60	≤0.001	10.10 ± 3.05	65.07 ± 2.48	≤0.001
	T1	52.87 ± 5.59	63.33 ± 2.60	≤0.001	34.21 ± 6.17	65.03 ± 2.49	≤0.001
	*p*	≤0.001	ns		≤0.001	ns	
	T2	61.47 ± 2.56	63.47 ± 2.53	≤0.001	51.69 ± 10.10	65.10 ± 2.45	≤0.001
	*p*	≤0.001	ns		≤0.001	ns	
	T3	62.27 ± 2.57	63.33 ± 2.60	≤0.001	64.44 ± 2.06	65.41 ± 2.21	≤0.001
	*p*	≤0.001	ns		≤0.001	ns	
Passive shoulder internal rotation	T0	13.67 ± 2.25	53.33 ± 2.60	≤0.001	13.14 ± 2.71	56.24 ± 2.92	≤0.001
	T1	51.27 ± 4.23	53.33 ± 2.60	≤0.001	33.76 ± 2.42	56.03 ± 2.74	≤0.001
	*p*	≤0.001	ns		≤0.001	ns	
	T2	52.20 ± 2.78	53.33 ± 2.60	≤0.001	51.86 ± 6.65	56.03 ± 2.74	≤0.001
	*p*	ns	ns		≤0.001	ns	
	T3	52.80 ± 2.95	53.33 ± 2.60	0.003	54.15 ± 3.23	56.44 ± 2.36	≤0.001
	*p*	ns	ns		≤0.001	ns	

**Table 2:** Comparison of the pain intensity assessment results obtained in the studied groups between the individual follow-up points.

Within-Group and Between-Group Comparison of Visual Analogue Scale [mm]			
	Group I	Group II	*p*
T0	47.20 ± 6.13	47.76 ± 6.07	Ns
T1	25.98 ± 2.59	23.86 ± 3.18	Ns
*p*	≤0.001	≤0.001	
T2	13.53 ± 1.68	14.28 ± 2.45	Ns
*p*	≤0.001	≤0.001	
T3	7.87 ± 3.42	6.44 ± 4.73	Ns
*p*	≤0.001	≤0.001	

Values expressed as arithmetic mean and standard deviation, ±. Group I, patients treated for a frozen shoulder by early arthroscopic capsular release, corticosteroid injection, and subsequent postoperative physiotherapy; Group II, patients treated for a frozen shoulder by corticosteroid injection and physiotherapy; *p*, level of significance; T0, just before the given treatment; T1, three months after T0; T2, six months after T0; T3, 12 months after T0.

**Table 3:** Comparison of the functional assessment results obtained in the studied groups between the individual follow-up points.

Within-Group and Between-Group Comparison of Quick DASH Score [*n* of Points]			
	Group I	Group II	*p*
T0	60.80 ± 9.63	61.93 ± 7.38	ns
T1	9.00 ± 8.03	51.55 ± 10.50	≤0.001
*p*	≤0.001	≤0.001	
T2	4.00 ± 3.81	25.00 ± 7.77	≤0.001
*p*	≤0.001	≤0.001	
T3	1.17 ± 2.52	4.22 ± 4.87	0.004
*p*	ns	≤0.001	

Values expressed as arithmetic mean and standard deviation, ±. Group I, patients treated for a frozen shoulder by early arthroscopic capsular release, corticosteroid injection, and subsequent postoperative physiotherapy; Group II, patients treated for a frozen shoulder by corticosteroid injection and physiotherapy; ns, not statistically significant; *p*, level of significance; T0, just before the given treatment; T1, three months after T0; T2, six months after T0; T3, 12 months after T0.

## Data Availability

The data presented in this study are available in Supplementary File named S1 Database.

## Supplementary Materials

The following are available online at https://www.mdpi.com/article/10.3390/jcm10215185/s1, S1: Database.
